# Editorial: HIV/HBV and/or HCV infections and hepatotoxicity in pregnant and non-pregnant women

**DOI:** 10.3389/frph.2025.1538380

**Published:** 2025-08-22

**Authors:** George Uchenna Eleje, Chinyere Ukamaka Onubogu, Emeka Philip Igbodike, Sayeed Mohammed Firdous

**Affiliations:** ^1^ Effective Care Research Unit, Department of Obstetrics and Gynaecology, Nnamdi Azikiwe University, Awka, Nigeria; ^2^ Department of Obstetrics and Gynaecology, Nnamdi Azikiwe University Teaching Hospital, Nnewi, Anambra State, Nigeria; ^3^ Department of Paediatrics, Nnamdi Azikiwe University, Awka, Nigeria; ^4^ Department of Paediatrics, Nnamdi Azikiwe University Teaching Hospital, Nnewi, Anambra State, Nigeria; ^5^ Department of Obstetrics and Gynecology, Kelina Hospital, Lagos, Nigeria; ^6^ Department of Pharmacology, Calcutta Institute of Pharmaceutical Technology & AHS, Howarah,-West Bengal, India

**Keywords:** co-infections, vaccination coverage, antiviral treatments, mother-to-child transmission (MTCT), HBsAg-positive

**Editorial on the Research Topic**
HIV/HBV and/or HCV infections and hepatotoxicity in pregnant and non-pregnant women

## Introduction

Viral infections such as human immunodeficiency virus (HIV), hepatitis B virus (HBV), and hepatitis C virus (HCV) remain major public health concerns globally, especially among women of reproductive age (1). Pregnancy introduces unique immunological and physiological changes that may influence the course of these infections, their management, and associated complications, including hepatotoxicity (1–3). This editorial unifies key findings from multiple studies to highlight how these infections intersect with maternal and non-maternal health outcomes, spanning pregnancy complications, treatment challenges, and innovative testing strategies in low-resource settings. The all-encompassing goal is to provide an interconnected understanding of how these infections impact women's health and identify gaps for future research and policy interventions.

## The need for integrated focus on HIV, HBV, and HCV in women

While previous studies has individually examined HIV, HBV, or HCV (4–6), few studies address their overlapping burden or combined implications for hepatotoxicity, especially across pregnant and non-pregnant populations (7–10). The four studies in this research topic illustrated in Table 1, collectively demonstrate that viral co-infections and associated liver complications require integrated screening, prevention, and treatment approaches. In all, they reinforce the necessity of linking maternal health interventions with broader women's health strategies.

**Table 1:** Summary of key findings from research studies on HIV, HBV, and HCV infections and hepatotoxicity in women.

Study	Population/setting	Focus/study topic	Key findings/results	Implications for maternal & women’s health
Weng et al. (China)	Pregnant women with HBV (HBsAg carriers)	Impact of HBV on pregnancy outcomes	- Persistent cervical shortening linked to higher risk of spontaneous preterm birth (HR = 2.00). - Lower risk of pregnancy-induced hypertension (PIH) and hyperthyroidism but higher risk of intrahepatic cholestasis of pregnancy (ICP). - Lower birth weight in HBsAg carrier group (aOR = 1.12).	Necessitates targeted prenatal care and routine HBV screening during pregnancy to mitigate complications.
Onambele et al. (Cameroon)	Pregnant women living with HIV in informal health centers	ART uptake and retention	- Only 34% initiated ART; median time to initiation was 7 days post-diagnosis. - 65.5% retained in care after 3 months. - Lack of self-efficacy strongly correlated with non-retention (aHR = 5.57). - Reasons for non-retention: fear of stigma, poor reception at referral centers.	Highlights gaps in continuity of care and emphasises need for policy reforms, improved counseling, and community-based support systems.
Ochwoto et al. (Kenya)	Antenatal care attendees	Integrated HIV, syphilis, HBV, and malaria testing panel	- Only 8.3% underwent all four tests; HIV positivity rate was highest (13.8%). - Introduction of integrated panel improved data management (91.7%) and reduced workload by 50%. - 4.5% tested positive for HBV.	Supports adoption of integrated testing panels to enhance maternal infection detection, reduce healthcare workload, and improve cost-effectiveness in low-resource settings.
Zhong et al. (China)	Postpartum women with chronic HBV infection	Pegylated interferon (Peg-IFN) therapy effectiveness	- Peg-IFN treatment resulted in 29.79% HBsAg loss at week 24 and 51.06% at week 48. - Seroconversion rate was 40.43%. - Higher proportion achieved undetectable HBV DNA at week 48 (95.24% vs. 71.43%). - Lower baseline HBsAg levels and postpartum flare predicted better outcomes (OR = 4.320).	Peg-IFN therapy appears safe and effective, offering a potential functional cure for chronic HBV in postpartum women and may be integrated into postpartum care strategies.

ART, antiretroviral therapy.

## Hepatitis B virus and pregnancy complications

The study by Weng et al. investigates how hepatitis B surface antigen (HBsAg) carriage affects pregnancy outcomes. This retrospective cohort study in Shenzhen, China, shows that HBsAg-positive women have lower risks of some complications, such as pregnancy-induced hypertension (PIH) and hyperthyroidism, but face higher risks of intrahepatic cholestasis of pregnancy (ICP) and delivering low birth weight infants. These findings emphasise the complex interplay between HBV and maternal health, highlighting the importance of risk-stratified prenatal care and ongoing screening.

## HIV management in informal health centers

Onambele et al.'s study examines the low uptake and retention of antiretroviral therapy (ART) among pregnant women living with HIV in informal health centers (IHCs) in Cameroon. Despite growing access to HIV screening, only 34% of women initiated ART, and retention after three months was 65.5%. This reinforces systemic gaps in continuity of care, pointing to the urgent need for policy interventions, enhanced counseling, and strengthened linkages between informal and formal healthcare settings.

## Integrating HIV, syphilis, HBV, and malaria testing in Kenya

Ochwoto et al. evaluated a four-test antenatal care (ANC) panel in Kenya, covering HIV, syphilis, HBV, and malaria. Their findings reveal how integrated testing significantly improved detection rates and streamlined service delivery, reducing healthcare worker burden while enhancing cost-effectiveness. This model demonstrates potential scalability for low- and middle-income countries seeking comprehensive maternal infection screening.

## Pegylated interferon treatment for hepatitis B in postpartum women

Lastly, Zhong et al.'s exploratory study highlights pegylated interferon alpha-2b (Peg-IFN) as a promising treatment for postpartum women with chronic HBV infection. The treatment achieved a 51.06% HBsAg loss rate and 40.43% HBsAg seroconversion rate after 48 weeks, with no serious adverse events. These results suggest that postpartum women may particularly benefit from Peg-IFN therapy due to immune reconstitution after pregnancy, offering a viable pathway toward functional cure.

## Conclusion

Together, these studies provide a comprehensive view of how HIV, HBV, and HCV infections intersect with hepatotoxicity and maternal outcomes in diverse settings. The unifying theme is the urgent need for integrated care approaches, of combining screening, prevention, and treatment, to address the complex burden of these infections across both pregnant and non-pregnant women. Strengthening health systems, bridging gaps between informal and formal care, and implementing cost-effective, mountable interventions are critical to improving outcomes and reducing disparities in low-resource settings. Future research must also explore long-term effects of maternal infections on both mothers and infants to inform sustained global health strategies.

Table 1 shows the summary of key findings from research studies.

The table summarises the main results related to HBV on pregnancy outcomes, ART uptake and retention in Cameroon, integrated testing in Kenya, and Peg-IFN treatment for HBV in postpartum women.
